# In Situ Thrust Measurement of Fish During Locomotion; Test Case: Sharks

**DOI:** 10.1002/ece3.71660

**Published:** 2025-06-27

**Authors:** Braedon Payne, Bryan A. Keller, Daniel Weihs, Roi Gurka

**Affiliations:** ^1^ Deprtament of Physics and Engineering Science Coastal Carolina University Conway South Carolina USA; ^2^ NOAA Fisheries Office of International Affairs, Trade, and Commerce Washington DC USA; ^3^ Faculty of Aerospace Engineering, Technion Haifa Israel

**Keywords:** fish locomotion, in situ, sharks, thrust

## Abstract

We present a novel method of measuring thrust of aquatic animals using in situ video data of swimming motions. To demonstrate its utility, the method was applied to several large elasmobranch species, which are typically highly challenging to measure. Using motion tracking software, we analyzed video footage of wild and captive sharks to track their instantaneous position and speed. In order to estimate the force output, we used the tail/body motion based on the swimming modes of the fish to calculate the water displaced by this motion during locomotion. Using Newton 3rd law, we have calculated the instantaneous force exerted by the water on the shark. The force output, that is thrust was calculated by averaging the instantaneous force over the tailbeat cycle. The thrust, for each fish was converted into a nondimensional parameter defined as: scaled thrust, allowing comparisons independent of size based on prior knowledge of the fish length and mass. This scaled thrust was analyzed for various swimming modes and caudal fin morphology to correlate to behavioral features through principal component analysis (PCA) we demonstrate the coupling between morphological traits and hydrodynamic forces. For the species studied the ratio of the upper to lower lobe of the caudal fin (CLAR) emerged as a strong predictor of scaled thrust, accounting for more than 80% of the observed variation. Our findings for the species studied indicate that coastal pelagic species exhibited lower scaled thrust values than benthic species, suggesting that benthic species may be less efficient, expending more energy to remain aloft or compensate for drag relative to generating forward motion. We propose that the unique ecological niches of these species drive behavioral changes that result in morphological adaptations to optimize performance.

## Graphical abstract

We present a novel method that uses in situ video data of fish locomotion to calculate thrust. This methodology was applied to several large elasmobranch species, which are typically challenging to measure. Our findings indicate that coastal pelagic species exhibited lower scaled thrust than benthic species, suggesting that benthic species may be less efficient.

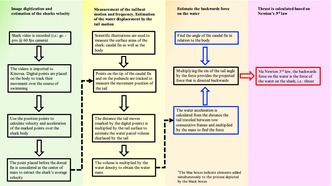

## 1 Introduction

Locomotion in nature is a key to survival (Alexander 2003). Almost all organisms move throughout their habitats. Locomotion varies as a function of the task and the medium (Biewener and Patek 2018). For example, most birds and insects fly, fish swim, and land animals run or walk. Fish and other aquatic animals need to generate thrust to counter drag when moving and to avoid sinking by generating lift or using some form of natural buoyancy, such as swim bladders. Regardless of the mechanism, these forms of movement require energy. Energetic considerations impact the ability of the animal to travel fast, cover distances, or carry a payload. We study how locomotive characteristics are associated within a range of fish within the subclass Elasmobranchii (sharks, and rays). We chose to focus on sharks and included batoids as the examples for our work due to the difficulties in measuring their motions in the wild. Shark species feature a diverse range of body shapes and life histories (Compagno 1990). The diversity of habitats in which they occupy created a range of shape specialization in order to perform optimally in their respective habitats, including their locomotive capabilities (Sternes and Shimada 2020).

The locomotive mechanisms of sharks have been extensively researched (Alexander 1965; Thomson and Simanek 1977; Flammang et al. 2011). A major key in the characterization of their locomotion is based on their swimming mode (Breder 1926). Swim mode is associated with a species behavioral ecology, including factors related to habitat use, spatial ecology, or predation strategies. Swim speed is associated with the energy expenditure (Blaxter 1980). Sharks feature morphological adaptations that serve to optimize their cost of transport (Ohlberger et al. 2006).

Hydrodynamic ability has been commonly characterized by classifying swimming patterns by body and fin motions (named “modes”) (Sfakiotakis et al. 1999). Breder (1926) defined four primary BCF swimming modes: anguilliform, subcarangiform, carangiform, and thunniform. Within this classification, a secondary division is made, separating locomotion by body and caudal fin oscillation (BCF) swimming, and Medial and Paired Fin (MPF) swimming. We deal with BCF swimming in the present study. Recent work by Di Santo et al. (2021) showed that the bounds of the modes originally proposed by Breder (1926) are somewhat fuzzy. However, they are still useful in generally clustering the species studied in regards to the thrust generation by different fractions of their body length. Each swim mode must also account for the recoil forces on the body caused by the lateral component of thrust. The tail plays a large role in reducing the recoil forces that act on the shark. As the tail transitions from movement of one side of the body toward the other, the tail angle rapidly changes direction to minimize lateral thrust. In anguilliform swimming, there is at least one entire propulsive wave present in the body. Therefore, anguilliform swimmers will actively counter the thrust in one direction of the body by the force on the other side of the body. Subcarangiform and carangiform swimmers counter recoil through a reduction in the body depth toward the peduncle and by concentrating the vertical cross‐section toward the anterior portion of the body. A similar approach is utilized by thunniform swimmers which have a streamlined body with optimized body shape and mass distribution (Sfakiotakis et al. 1999).

Morphological features, such as the caudal fin, play a role in generating the hydrodynamic forces required for locomotion (Flammang et al. 2011). The caudal fin is commonly characterized by its aspect ratio, which is defined as the squared surface area of the tail divided by height. This is used as a measure for the propulsive ability of each species. Sumikawa et al. (2023) performed numerical analysis on the caudal fin morphologies of multiple shark species. They found that caudal fins with a large aspect ratio had higher thrust and swimming costs, whilst small ratios had higher propulsive efficiency. An additional parameter‐ caudal lobe aspect ratio (CLAR), describes the size ratio of the upper lobe to the lower lobe (Iliou et al. 2023). Iliou et al. (2023) demonstrated that shark species with low CLAR tended to be faster moving and have higher average speeds. Chu et al. (2025) found that for symmetrical tails (i.e., white shark), the CLAR was correlated with the caudal vortex shape and pressure difference, affecting the thrust generated and lateral force coefficients. In addition, this parameter is helpful to describe how much lift a shark is able to produce while swimming. For example, a heterocercal tail is essential for generating lift and maintaining position in the water column (Wilga and Lauder 2002); this is accomplished due to the size of the lower lobe relative to the upper lobe. Other morphological characteristics, such as body shape, are also variable based on swim mode. Sternes and Shimada (2020) found that sharks that are shallow bodied tend to inhabit benthic habitats, exhibit anguilliform motion, and possess low aspect ratio tails. Deep bodied sharks are known to inhabit pelagic waters, exhibit thunniform and carangiform motion, and feature high aspect ratio tails.

We seek to demonstrate the variations in hydrodynamic output between sharks by characterizing their tail motion through the use of high‐speed camera footage. Considering their behavioral ecology, swim mode, tail morphology, and genetic relatedness, we identify some of the factors that influence or associate with their swim mode. Evaluating the momentum (i.e., forces) of sharks during locomotion has helped provide insights toward how they balance energy consumption with other functions that are necessary to their survival.

## 2 Methods

### 2.1 General Approach

High speed cameras were used to collect footage of swimming sharks. The videos were exported to Kinovea (www.kinovea.org), a motion tracking software, to track the position of different points on the shark over a set time period. Displacement, velocity, and acceleration of certain points were used in conjunction with the tail surface area to estimate the force output. Each thrust value was scaled based on mass, frequency, and velocity in order to compare between different sizes of sharks.

### 2.2 Shark Species Selection

We used high‐speed images that were processed in order to analyze the hydrodynamics of sharks during straight‐line swimming. The images were acquired for several shark species (Table 1). We also captured the swimming of the largetooth sawfish ( *Pristis pristis* ), a batoid, which serves as a comparison of how hydrodynamic output efficacy has evolved. Footage for the tiger shark ( *Galeocerdo cuvier* ), white shark ( *Rhincodon typus* ), great hammerhead (*Sphyrna mokorran*) and zebra shark (*Stegostoma tigrinum*) was collected via drone imaging. The remaining species were monitored at the National Aquarium, Baltimore, MD, USA. Table 1 summarizes the species studied with their average length, weight, family, and swim mode.

**TABLE 1:** The shark species measured within this study, including length, weight, family, and swim mode^a^.

Species	Length (m)	Weight (kg)	Family	Swim mode
Largetooth sawfish *Pristis pristis*	3.2	142.5	Pristidae	Carangiform
Nurse shark *Ginglymostoma cirratum*	2.6	105.9	Ginglymostomatidae	Subcarangiform
Zebra shark *Stegostoma tigrinum*	2.3	71.5	Stegostomatidae	Subcarangiform
Blacktip shark *Carcharhinus limbatus*	1.2	12.6	Carcharhinidae	Carangiform
Blacknose shark *Carcharhinus acronotus*	1.2	10.5	Carcharhinidae	Carangiform
Bull shark^b^ *Carcharhinus leucas*	2.5	335.7	Carcharhinidae	Carangiform
Tiger shark^b^ *Galeocerdo cuvier*	2	78	Galeocerdonidae	Carangiform
Sand tiger shark *Carcharias taurus*	2.4	89.3	Odontaspididae	Carangiform
Sandbar shark *Carcharhinus plumbeus*	2	58.6	Carcharhinidae	Carangiform
Great hammerhead^b^ *Sphyrna mokorran*	3.1	325	Sphyrnidae	Subcarangiform
Whale shark^b^ *Rhincodon typus*	7.6	18,688	Rhincodontidae	Carangiform
White shark^b^ *Carcharodon carcharias*	4.7	1346	Lamnidae	Thunniform

^a^ Maia et al. (2012); Sternes and Shimada (2020).

^b^ Sharks marked with an asterisk identify videos sourced from the wild using drone footage.

### 2.3 Imaging Collection

**FIGURE 1:**
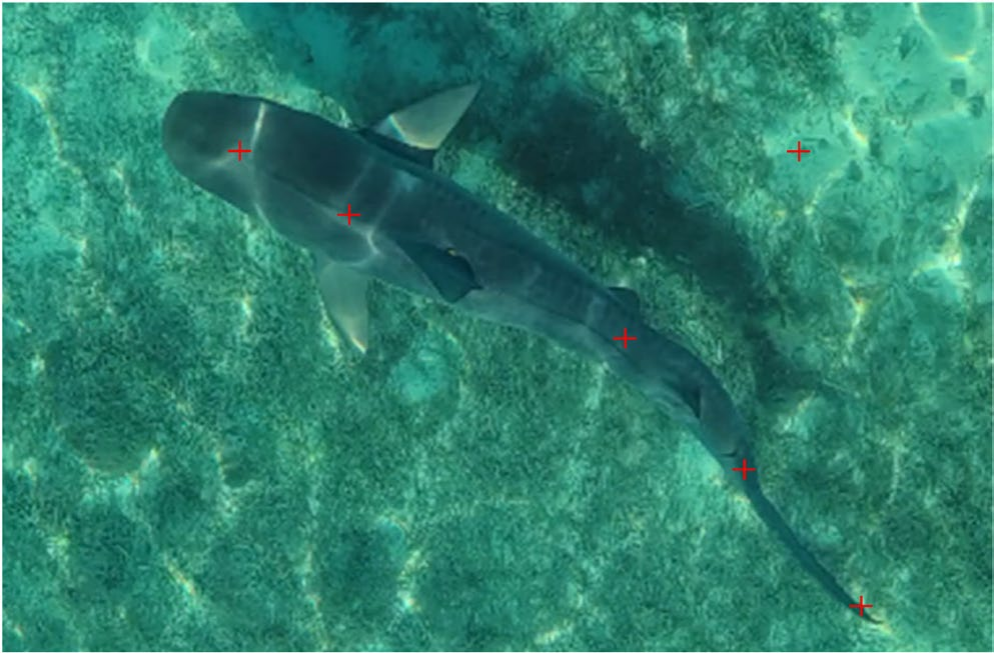
An example of shark footage (showing a tiger shark ( *Galeocerdo cuvier* )). The image was extracted from one of the videos recordings (images taken by Dr. Craig O'Connell and Chelle Blais) depicting shark swimming. The red cross markers depict five points placed along the body including one point placed on the sea floor to use as a reference.

The range of species covers variation in length, mode of locomotion, swimming speed, and tail beat frequency. The videos recorded in the National Aquarium were acquired by a GoPro camera operating at 60fps and 4 k resolution, positioned perpendicular to the dorsal side of the body such that the plane of the camera was parallel with the motion (Figure 1). Each recording session lasted at least 45 min to passively capture the movements of sharks through the tank. The Research Committee of the National Aquarium reviewed and approved the described methodology in August 2023. As this study was purely observational in nature and required no direct contact or interference with the involved animals, no additional ethics approvals were required.

The drone footage was captured by flying the drone over a swimming shark, with the camera pointed directly downwards (video captured by Dr. Craig O'Connell (https://www.oseasfdn.org/) and Chelle Blais: (https://www.chelleblais.com)). If the drone was moving to track the shark's movement, we ensured that there were fixed geographic features present to compare to the motion. Drone footage was obtained from online resources or acquired directly from the operators; we used the technical details provided within the videos scripts or estimated some of the technical parameters based on catalog information, for example the camera frequency and resolution at 30 or 60fps and 1080p. We then extracted occurrences in which the sharks swam underneath the camera. On average, 3 videos per shark specimen were analyzed with high image quality, yielding 3 cycles of tail/body motion generating thrust at constant speed. The videos were decomposed into digital images based on the camera frequency.

For the sharks that were observed at the National Aquarium, length and weight were provided. Different criteria were used to determine these values for shark video gathered by drones. The drone footage taken of the white shark and bull sharks was accompanied by a length that was estimated in reference to nearby geographic features that appeared in the image with prior known size by the videographer. This length was referenced to a length‐to‐weight table for various shark species, and the described mass for their length was used. Other species that did not have accompanying lengths or weights provided were treated as an average adult of the species, and the average length and weight were used in the analysis (Kohler et al. 1996). While some values may not be accurate, either due to lack of precision in our estimates or individual variation in weight at specific lengths, these factors impose limitations on one another (see the *Thrust Estimation* section for a discussion on thrust calculation for more details).

Using Kinovea (www.kinovea.org), multiple points along the fish body were tracked for at least one period of the tail beat. The points were placed on the head, pre‐dorsal, post‐dorsal, peduncle, and caudal fin (a total of 5 points for each animal). The resulting path trajectories in 2D (i.e., x, y corresponding to streamwise and spanwise coordinates in a global fixed coordinate system) as well as marked points on the tail enabled tracking the motion during swimming. Knowing the camera frequency, the fish speed can be obtained, following image calibration. Based on these trajectories, we calculated the shark speed, thrust, and the tail frequency simultaneously. Each cycle lasted several seconds, signaled by when the tail returned to its original starting place.

### 2.4 Thrust Estimation

**FIGURE 2:**
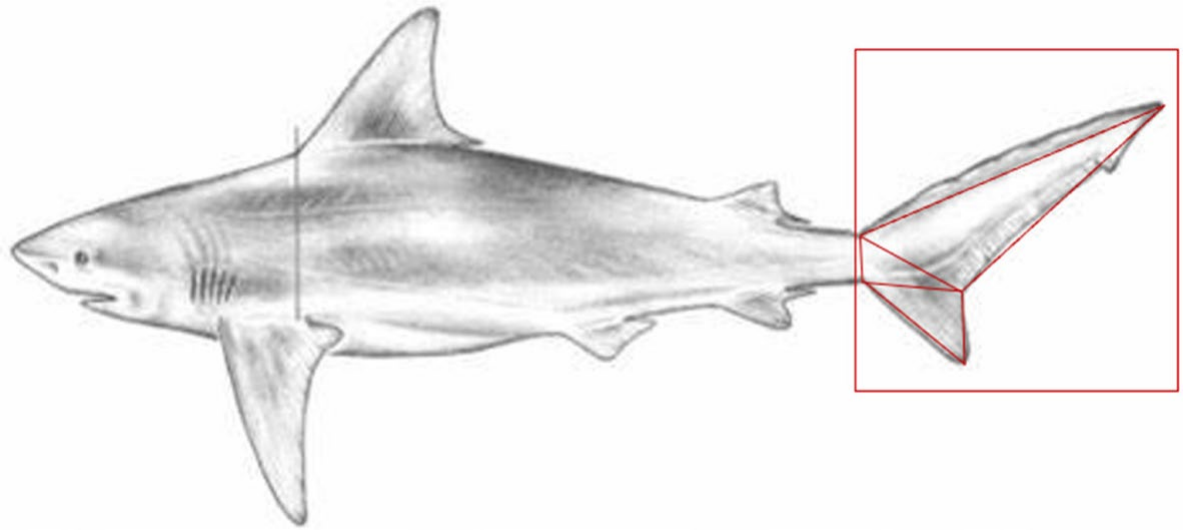
Diagram showing the tail surface area estimation from the bull shark ( *Carcharhinus leucas* ) (adapted from Grace (2001)).

We measured instances when sharks were swimming at constant speed over at least 3 cycles of tail/beat motion. This means there was no apparent acceleration; thus, no force could be extracted from the speed. We seek to find the force of the animal on the water i.e., thrust, which is equal to the force of the water on the animal. In order to obtain the force, we calculated the momentum of the displaced water by the shark between each frame. The thrust is generated through the body undulation (Lighthill 1969) hence, the water is displaced as a function of the tail/body movement (Drucker et al. 2005). A conservative approach is established by marking a parcel of water that is displaced by the motion of the tail (in a linear fashion on the tail coordinate system (Webb and Weihs 2015)). Herein, the water bulk is assumed to be moving as a solid body where the total force is distributed throughout the surrounding water. Therefore, an estimation of the water bulk volume is required. The distance the tail travels between each frame is used as the width of the water bulk that varies over time during locomotion. This width multiplied by the normal area will provide a measure of the volume. This is done by using scientific illustrations of each shark (Grace 2001) and pixel measuring software to measure the surface area of the caudal fin. Three triangles were created to measure the upper lobe, lower lobe, and peduncle (see for example Figure 2). The length of the sides of each triangle was scaled by the pixel‐to‐length ratio of the shark to extract real size. Multiplying length, width, and height together results in the volume of the displaced water, and factoring by the density of seawater yields the mass. Treating the water bulk as a solid body that is displaced; thus, accelerates when the tail travels through gives the acceleration of the water, which can then be multiplied by the mass to extract the force. The angle of the tail in relation to the direction of motion is used to calculate the component of force directed backward (Webb and Weihs 2015), since the side‐to‐side thrust cancels out over a full cycle as the tail returns to its starting configuration.

**FIGURE 3:**
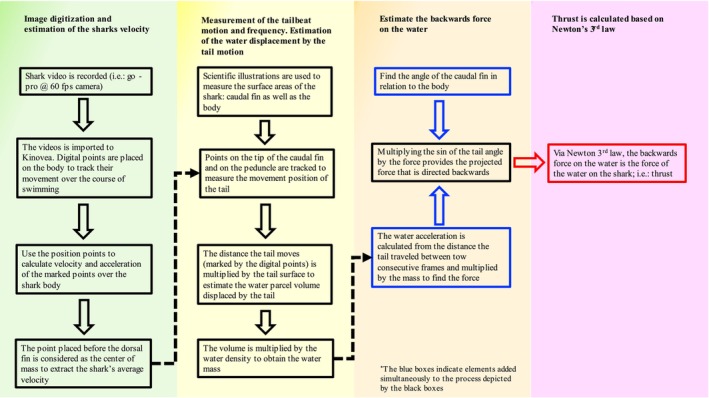
Flowchart illustrating the process for obtaining thrust from video footage is described. Green: Image acquisition and motion tracking is initialized. Yellow: Initial steps of analysis for calculating thrust. Orange: Compilation of variables to obtain backward force vector. Pink: Application of Newton's 3rd law to obtain thrust. Dark blue rounded boxes were added to signify the addition of new factors with a dotted line to symbolize the relationship between the two boxes. The red box is added to reflect the final value.

Figure 3 describes conceptually how the thrust was calculated from the images. The process is comprised of 4 stages: (i) image acquisition and markers identification for each shark, (ii) kinematic analysis of the markets to deduce speed and direction, (iii) estimation of the reactive force by the water due to the tail movement and (iv) thrust estimate. Step (i) follows the basics of using 2D imaging to extract the kinematics of moving objects using Kinovea and step (iv) is an algebraic calculation of the properties calculated in steps (ii) and (iii). Detailed description of steps ii and iii, which are the extraction of the kinematics values and the thrust estimates based on the momentum equilibrium between the shark and the surrounding water are provided in Table 2, where we provide the algebraic path to estimate forces from images during locomotion.

**TABLE 2:** Thrust estimation procedure based on the measured kinematics.

Objective	Function
Velocity/acceleration Center of mass	Find *Δx* between each frame for the *X* and *Y* coordinates. *Δx* is the distance traveled marked points between consecutive frames 2 Calculate velocity: V=∆x/t 3 For acceleration: a=∆V/t *ΔV* is the velocity difference of marked points between consecutive frames
Tail oscillation	Estimate the lateral velocity of the tail: W=0.2πLf, (Webb and Weihs 2015) *L* is the fish length and *f* stand for the frequency of the tail beat Subtract center of mass position and find *Δx* between each frame; ∆x=W×t Use tail motion to estimate the acceleration of the water that is displaced by the tail: $a=2∆x/t^{2}$ (*t* is the time interval between consecutive images) Calculation of the water volume displaced by the tail: $m=\rho \Delta x\left(\mathrm{SA}\right)$ is the water density, dx is the distance of marked points at the tail region traveled along time and *SA* is the tail surface area Force generated by the tail: $F^{\prime }=\mathrm{ma}$ repeat Steps 2–5, substituting the peduncle for the tip of the tail Calculate the average force of the tail for the two points The relative force on the water that is directed backwards: $F=F^{\prime }\sin\theta$ is the tail angle of orientation Newton's third law: $T_{\text{shark thrust}}=F$
Tail angle $\left(\theta \right)$	Tail angle: Subtract *X* and *Y* coordinates of caudal peduncle by the coordinates of the tip of the tail at each frame Body angle: Subtract *X* and *Y* coordinates of a point placed in front of the dorsal fin by the point placed behind the dorsal fin Determining the direction of motion: Subtract the Tail Angle by the Body Angle to compare the difference between the angle of the tail and direction of motion
Thrust coefficient	The thrust coefficient is found using the previously described thrust force normalized by the mass, its velocity and tail frequency: $C_{T}=T/\mathrm{mVf}$

## 3 Results

The thrust was normalized in order to allow comparison between the species. It was normalized by the mass, *m*; tailbeat frequency, *f*, and the shark's speed, *V*:

(1) $$C_{T}=T/\mathrm{mfV}$$

yielding a non‐dimensional scaled thrust (Table 3). The mass of the shark was used to account for the size and muscle mass required to move it. The tail beat frequency was used to limit the influence of the rate of the tail's movement and additional energy expenditure by the shark, and the velocity of the shark to account for variation in speed across species. The resultant value provides insight on the individual force expenditure of the shark and can be used to further discuss their hydrodynamic performance. We performed the same analysis on a video collected of a zebra shark swimming in the wild and in ex‐situ measurements taken at the National Aquarium. In the wild (based on the drone videos), the scaled thrust value was 8.94, which is elevated compared to the value of 5.76 from the aquarium; while some variation is present, these values are still much higher than the average observed across the other species. This comparison supports our assumption that the observed behavior in the aquarium is a good proxy for swimming behavior in nature (see Appendix S1 for calculation of voluntary swimming speeds extracted from the data based on Weihs et al. (1981)).

**TABLE 3:** Thrust output and thrust scaling of the shark species.

Shark	Thrust (*N*)	Velocity (m/s)	Weight (kg)	Frequency (Hz)	Scaled thrust	Sample size
Largetooth sawfish	542.2	1.13	142.5	0.47	0.69	1
Nurse shark	227.6	1.05	105.9	0.32	4.19	3
Zebra shark	194.2	0.87	71.5	0.54	5.76	1
Zebra shark^a^	301.4	0.50	71.5	0.25	8.94	1
Blacktip shark	11.64	0.96	12.6	1.1	1.1	10
Blacknose shark	4.75	0.59	10.5	1.05	0.72	3
Bull shark^a^	75.6	0.84	335.7	0.5	0.51	3
Tiger shark^a^	17.5	0.70	78	0.33	1.03	3
Sand tiger shark	25.9	0.54	89.3	0.4	1.14	3
Sandbar shark	20.5	0.96	58.6	0.56	0.66	7
Great hammerhead^a^	183.6	0.48	325	0.42	0.8	3
Whale shark^a^	2048	1.31	18,688	0.21	0.42	1
White shark^a^	784.5	1.34	1346	0.41	1.07	2

^a^ Sharks marked with an asterisk to identify their thrust estimate are based on videos sourced from the wild captured (aerial imaging).

**FIGURE 4:**
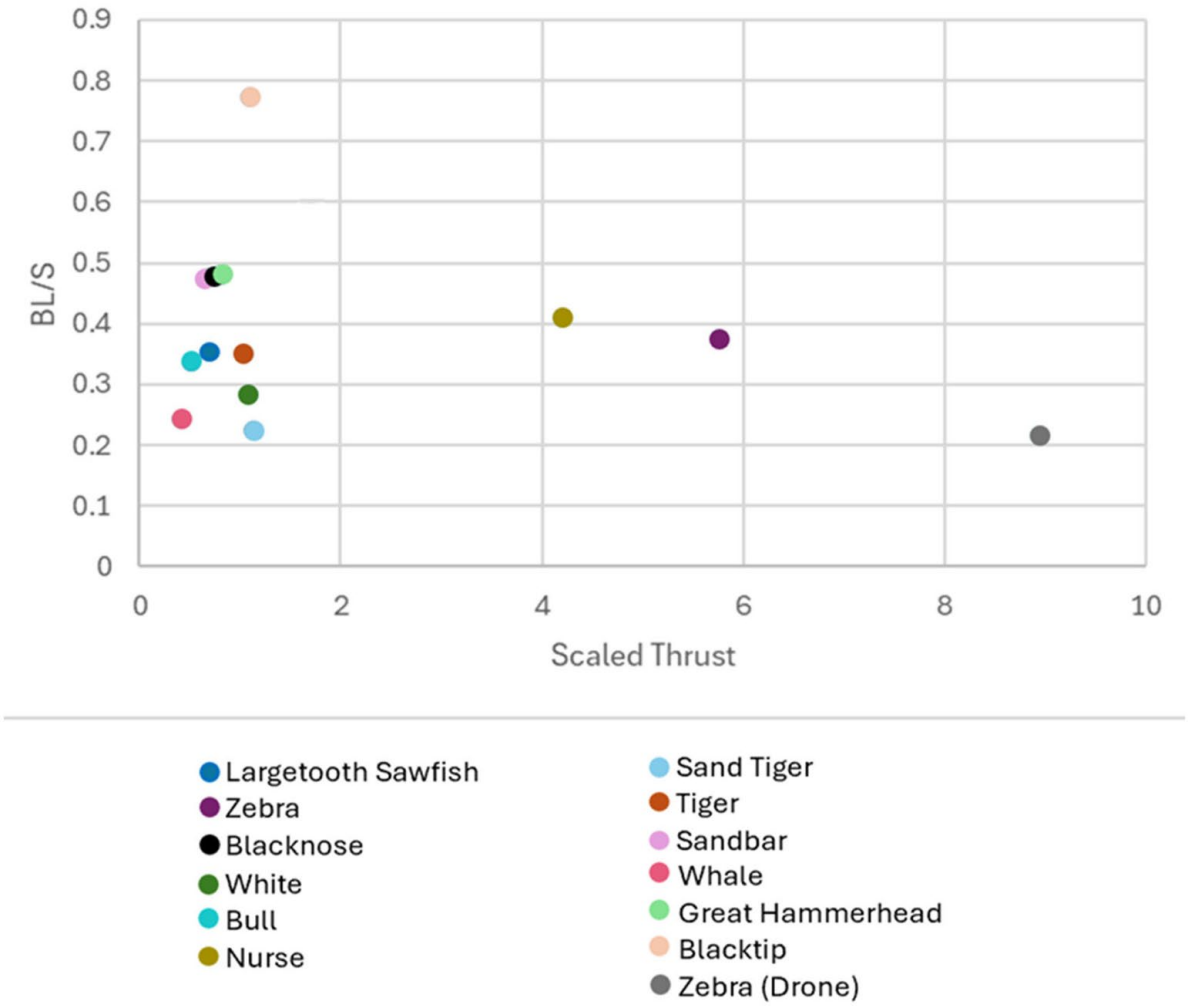
Average scaled thrust measured alongside velocity for each species. All species tested were sharks aside from the largetooth sawfish.

A higher scaled thrust value means that a shark is producing more thrust in relation to its body mass and its tail beat frequency. However, we have yet to demonstrate how the shark's scaled thrust value impacts its velocity. The scaled thrust was plotted alongside the average body lengths per second to identify how scaled thrust values may translate into forward body motion in Figure 4.

Figure 4 demonstrates that the three sharks with higher scaled thrust are moving within the same range of velocity (0.2–0.4 BL/S) as the other sharks (0.2–0.8) BL/S). It appears that sharks with higher scaled thrust values are less efficient at translating tail motion to forward body motion because having a higher scaled thrust value did not result in increased body speed (Figure 4). The three points that appear apart from the others (scaled thrust between 4 and 10) are the zebra and nurse sharks. For the zebra shark, both instances from the aquarium and from drone footage each resulted in values outside of the main cluster (see scaled thrust values in Table 3). Both sharks are considered to be benthic and, thus they presumably spend more energy to staying away from the bottom or compensating for drag while moving and this likely contributes to inefficiency observed here in respect to the scaled thrust.

**FIGURE 5:**
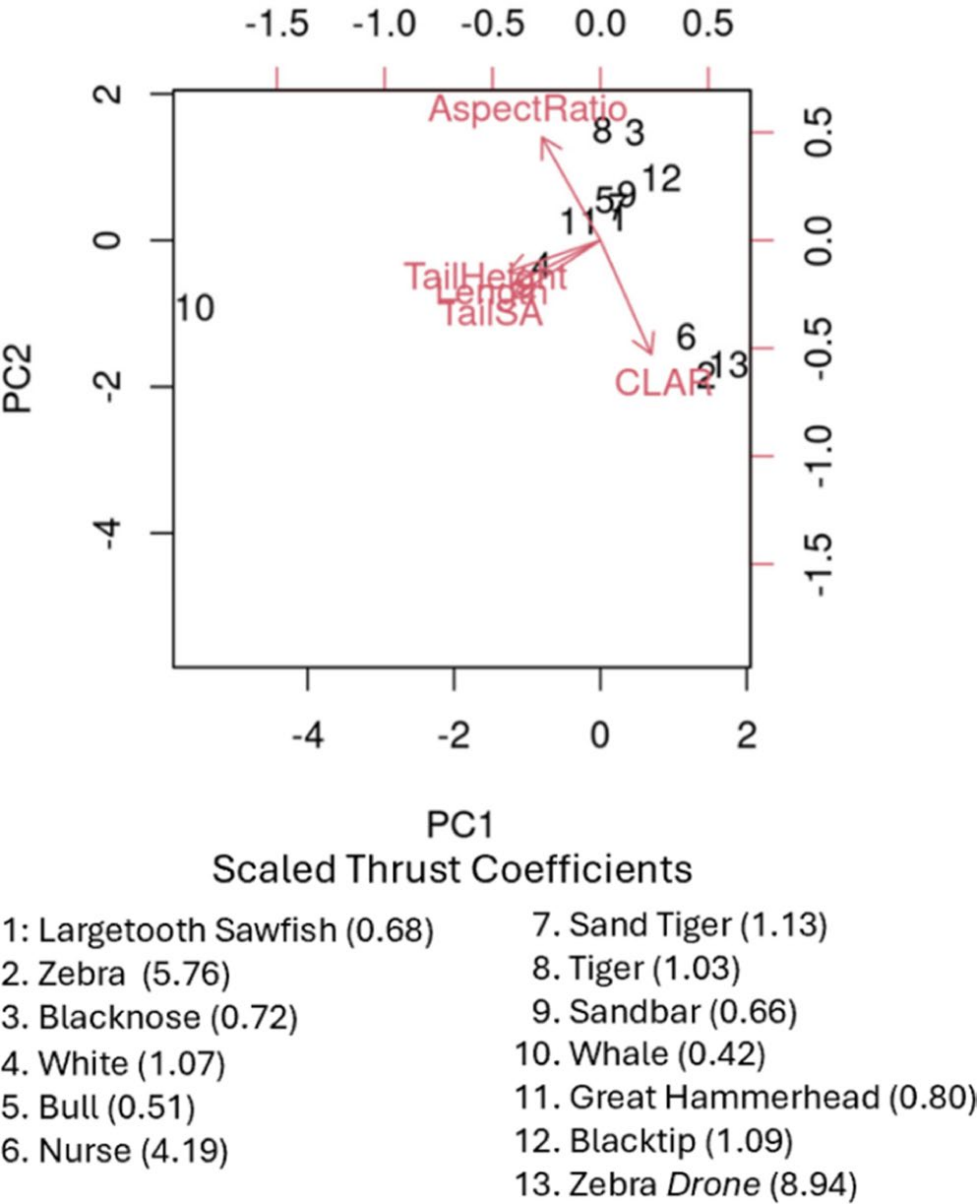
Principal components analysis between species based on morphological features. All species tested were sharks aside of the largetooth sawfish.

In order to assess this result, we applied PCA to four morphological features of the shark caudal fin: height, length, tail surface area, aspect ratio (AR) and upper to lower lobe ratio (CLAR) and the estimated thrust as shown in Figure 5. Based on Figure 5, most of the sharks were clustered in the top right of the chart. The whale shark (10) was located on the far‐left side of the chart. Tail height, length, and surface area are not standardized measurements, which could explain the observed variability due to the whale shark's length. The instances labeled ‘2’ (zebra), “6” (nurse), and “13” (zebra: drone) were each located near the CLAR label. Not only do these two species differ from the other sharks based on their scaled thrust output, they also differ from the other species in CLAR. Based on the PCA (see variance explained in Table 1 in Appendix S1), we identified that there may be a relationship between CLAR and Scaled thrust. Pearson's correlation returned a value of 0.91, which indicates a strong positive correlation between these two factors, *r* (11) = 0.91, *p* = 1.71e^−05^.

## 4 Discussion and Conclusion

We suggest an approach to estimate In situ thrust during fish locomotion. This is demonstrated in elasmobranch fishes, with a focus on sharks. We focus on sustained swimming (constant speed), excluding acceleration, bursting, or maneuvering swimming, which require a lot of power and energy over a short time interval. All these are associated with Body‐Caudal Fin (BCF) motion (Webb 1984; Blake 2004). Thus, thrust is produced by undulating certain portions of the body (Lauder 1989). The motion is achieved by displacing water by the body undulations, as described above. Hence, the hydrodynamics are a result of the force balance between the displaced water and the moving parts of the body (using Newton 3rd law). We demonstrate the feasibility of the method by applying it to shark locomotion. The method is applied to video recordings of sharks in the wild and in a controlled environment (i.e., aquarium). The video recordings are extracted into sequences of digital images that are analyzed to obtain the sharks kinematics, whilst physical dimensions (i.e., length and weight) of the species need to be estimated prior to the thrust calculations.

The advantage of this method in comparison to other methods such as equipping accelerometers on marine species (Gleiss et al. 2011) or using satellite tagging (Sleeman et al. 2010) is that these approaches are costly, incorporating logistical challenges such as retrieval as well as facing ethical constraints associated with fish stress and health. Recently, Gharabaghi (2025) used video images to track whale sharks and estimated their swimming speed, but no hydrodynamic force estimate was presented; here, we provide a unique approach to further analyze video images to extract these forces.

The species tested, range over 3 modes of locomotion: thunnifom (number of species *n* = 1), subcarangiform (*n* = 3), and carangiform (*n* = 8). The scaled thrust appears to be correlated with the modes of locomotion. As shown in Table 3, the range of scaled thrust for subcarangiform swimmers is much higher than both carangiform and thunniform swimmers. However, it appears that behavioral ecology and morphology may be more impactful than swimming mode. For example, the benthic species (nurse and zebra shark) had higher scaled thrust than the coastal pelagic (narrow differences in scaled thrust between 0.4 and 1.2). Although one may hypothesize that this variation is connected to phylogenetics as the species are closely related, the values for the whale shark do not support this theory. The whale shark, which is in the same order (Orectobliformes) as nurse and zebra sharks has a scaled thrust value (0.42) that lies within the previously outlined range. We looked for other explanatory factors that could explain the variation in our study. It is worth noting that the scaled thrust of the largetooth sawfish (0.69), a batoid and near relative of sharks fell within the range for coastal pelagics (0.4–1.2). The largetooth sawfish has similar body motion and tail morphology to the sharks within this group, so it is not surprising that their measurements were similar. This supports the notion that variabilities in hydrodynamics are driven, at least in part, by behavioral ecology and morphology. The observed narrow range of hydrodynamic output amongst coastal pelagic species suggests that regardless of the family classification, the most dominant factor influencing scaled thrust is the sharks' behavioral ecology, whether they are benthic or coastal pelagic swimmers, and the related effects on morphology (Sumikawa et al. 2023). In order to assess this result, we applied PCA (principal component analysis) to four morphological features of the shark caudal fin: height, length, aspect ratio (AR) and upper to lower lobe ratio (CLAR) as shown in Figure 5. The Pearson's correlation shows a strong relationship between CLAR and scaled thrust, similar to observations by Iliou et al. (2023) and Chu et al. (2025). Essentially, the CLAR accounts for over 80% of the observed variation in scaled thrust, as defined by the *R* ^2^ value. While the behavioral ecology of the species affects the scaled thrust, the tail morphology is the physical trait which drives the thrust. As the lower lobe becomes smaller relative to the upper lobe, the animal devotes more energy to generating lift and becomes less effective in producing forward body motion.

An additional outcome is that the scaled thrust correlates well with the CLAR. The zebra and nurse sharks demonstrate how physical considerations impact the morphological structure of the fin to comply with its hydrodynamical tasks: for these benthic species, the required lift is smaller compared to the thrust generation as they can take advantage of ground effects (Quinn et al. 2014). Furthermore, the association of a morphological parameter: CLAR appears to better correlate with the thrust compared to the other three. This suggests that the shark morphological shapes evolved to correspond to physical constraints based on their ecological tasks in order to optimize their energy expenditure. These geometrical dependencies on the thrust indicate strong coupling between the biological and physical considerations.

We hope this research can be expanded to other groups of fish, such as other shark families like lamnids with nearly homocercal tails, in order to continue developing our understanding of how morphology can affect swimming efficiency.

## Author Contributions

**Braedon Payne:** data curation (lead), formal analysis (lead), investigation (lead), methodology (equal), validation (lead), writing – original draft (equal). **Bryan A. Keller:** data curation (supporting), formal analysis (supporting), investigation (equal), methodology (supporting), writing – original draft (equal), writing – review and editing (equal). **Daniel Weihs:** conceptualization (equal), methodology (supporting), writing – original draft (equal), writing – review and editing (equal). **Roi Gurka:** conceptualization (lead), investigation (equal), methodology (equal), writing – original draft (equal), writing – review and editing (equal).

## Conflicts of Interest

The authors declare no conflicts of interest.

## Supporting information


Appendix S1.

## Data Availability

All the data is uploaded as Supporting Information.
