# Supplementary material for: In Situ Thrust Measurement of Fish During Locomotion; Test Case: Sharks
Source: Ecol Evol. 2025 Jun 27;15(7):e71660. doi: 10.1002/ece3.71660 (PMC12203120; doi:10.1002/ece3.71660)
Supplement: Supplementary file 1 — Appendix S1. [file ECE3-15-e71660-s001.zip › appendix_a1_pca_analysis_shark_locomotion.docx]

Table 1: Variance explained for the PCA analysis performed over the tail sharks morphology in respect to the thrust

| Tail morphology | PC1 | PC2 | PC3 | PC4 | PC5 |
| --- | --- | --- | --- | --- | --- |
| Tail SA | -0.5077283 | -0.3342605 | 0.08438951 | 0.4867370 | -0.62164894 |
| Length | -0.5053886 | -0.2552798 | -0.37414365 | -0.7308256 | -0.07297301 |
| Tail height | -0.5343120 | -0.1820692 | 0.21197494 | 0.2488899 | 0.75794586 |
| Aspect ratio | -0.3381650 | 0.5965864 | 0.64417057 | -0.2862212 | -0.18124788 |
| CLAR | 0.2948719 | -0.6588153 | 0.62690178 | -0.2917401 | -0.02991333 |
